# Direct medical costs of young-onset colorectal cancer: a worldwide systematic review

**DOI:** 10.1186/s12913-022-08481-6

**Published:** 2022-08-30

**Authors:** Ria Garg, Vicki Cheng, Ursula Ellis, Vanay Verma, Helen McTaggart-Cowan, Stuart Peacock, Jonathan M. Loree, Mohsen Sadatsafavi, Mary A. De Vera

**Affiliations:** 1 Faculty of Pharmaceutical Sciences, University of British Columbia, Vancouver, Canada; 2 Collaboration for Outcomes Research and Evaluation, Vancouver, BC Canada; 3 University of British Columbia Library, Vancouver, BC Canada; 4 BC Cancer, Vancouver, BC Canada; 5 Faculty of Health Sciences, Simon Fraser University, Burnaby, BC Canada; 6 Faculty of Medicine, Department of Medicine, Division of Medical Oncology, University of British Columbia, Vancouver, BC Canada; 7 Centre for Health Evaluation and Outcome Sciences, Vancouver, Canada

**Keywords:** Young-onset colorectal cancer, Costs, Systematic review

## Abstract

**Background:**

Given the rising incidence of young-onset colorectal cancer (yCRC) among individuals younger than 50 years old, understanding the economic burden of yCRC is required to inform the delivery of healthcare services. Therefore, we conducted a systematic review of studies assessing the direct medical costs of yCRC, and where relevant average-age onset CRC (aCRC).

**Methods:**

We searched MEDLINE, EMBASE, and Web of Science from inception to May 2022 for original, peer-reviewed studies, that reported direct medical costs (e.g., chemotherapy, radiotherapy, outpatient visits, inpatient care, prescription medications) for yCRC and aCRC. We used a modified version of the Consolidated Health Economic Evaluation Reporting Standards checklist to appraise the studies. Costs were inflation-adjusted to 2020 US dollars.

**Results:**

We included 14 studies from 10 countries, including the USA, England, France, Korea, Vietnam, China, Italy, Australia, Canada and Japan. Five studies focused on prevalent disease and reported annualized per-capita cost of prevalent yCRC, ranging from $2,263 to $16,801 and $1,412 to $14,997 among yCRC and aCRC cases, respectively. Nine studies estimated the cost of incident disease. Synthesis of per-capita costs incurred 12 months following colorectal cancer diagnosis ranged from $23,368 to $89,945 for yCRC and $19,929 to $67,195 for aCRC. Five studies used multivariable approaches to compare costs associated with yCRC and aCRC, four showed no differences and one suggested greater costs with yCRC.

**Conclusion:**

Our synthesis of direct medical costs of yCRC across multiple jurisdictions provide relevant information for healthcare decisions, including on-going considerations for expanding CRC screening strategies to younger adults.

**Supplementary Information:**

The online version contains supplementary material available at 10.1186/s12913-022-08481-6.

## Introduction

Colorectal cancer (CRC) is the third most common malignancy and the second most deadly cancer worldwide [1]. With respect to CRC, in 2020 alone there were an estimated of 1.9 million incident cases and 0.9 million deaths reported worldwide [2]. While the risk of CRC is the highest in developed countries, middle- and low-income countries have also reported an increasing trend in the incidence of CRC, which may be due to the adoption of different lifestyle choices, such as decreased physical activity and diet modifications [1]. Although CRC is traditionally considered a disease in older adults, with average age of onset at 50 years or older (aCRC), research over the past decade has shown a rise in the incidence of young-onset colorectal cancer (yCRC) across the world – that is, CRC occurring in individuals younger than 50 years [3]. Specifically, a 2019 cohort study which explored increasing yCRC incidence in various countries around the globe, reported a greater annual percent change in incidence among yCRC versus aCRC in countries such as Australia (+ 1.10% vs. -0.35%), Brazil (+ 9.20% vs. + 5.72%) and Japan (+ 2.63% vs. + 0.90%) [4].

With the increasing risk of yCRC, comes the need for understanding its economic impact; of particular interest are direct medical costs – costs that are directly related to provision of medical services for patient care (i.e., prescription medications, inpatient care, outpatient visits, chemotherapy, radiation therapy). Additionally, individuals with yCRC are more often diagnosed at later stages and with metastatic disease in comparison to individuals with aCRC, largely due to the lack of age-specific diagnostic guidelines [5]. As a result, yCRC patients tend to receive more aggressive treatments, particularly those involving multi-agent systemic chemotherapy and local irradiation, which may lead to higher costs overall [6]. However, it is unclear whether direct medical costs of yCRC have been evaluated. As such we conducted a systematic review to: 1) understand how the economic burden of yCRC has been evaluated (e.g., are there specific studies; are there studies of CRC that also include those with yCRC; what age cut-off has been used to define yCRC); 2) to synthesize reported direct medical costs of yCRC; and 3) where relevant, compare direct medical costs between yCRC and aCRC.

## Methods

### Search strategy

We conducted a literature search of Ovid MEDLINE, Ovid Embase and Science Citation Index and Social Sciences Citation Index via Web of Science from inception to July 15, 2021, and then updated the search on May 30, 2022. To ensure comprehensive capture of articles that may assess yCRC as a subgroup of CRC, we employed a broad search strategy to identify articles on CRC across all ages, from which data pertaining to yCRC could be extracted. Our search strategies used a combination of subject headings (e.g. Medical Subject Headings in Medline) and keywords to locate studies. Search terms related to economics/costs were adapted from a search filter developed by Canadian Agency for Drugs and Technologies in Health [7] (Tables S1-3).

### Study selection

We reviewed titles and abstracts to identify published studies that met our systematic review inclusion criteria of: 1) an original study; 2) published in a peer-reviewed journal as a full-length article; 3) patient population with CRC or yCRC; 4) published in English; and 5) reported the direct medical cost of yCRC, defined as costs directly related to provision of medical services for patient care (e.g., surgery, prescription medications, inpatient care, outpatient visits, chemotherapy therapy and radiation therapy). Given potentially different age cut-offs for defining ‘young-onset’, for the purposes of our systematic review, we broadly considered an age cut-off of up to 65 years old in their cost estimation and reported as yCRC. We excluded non-original literature (i.e., reviews and editorials), economic evaluations (i.e., cost-effectiveness of interventions or programs), studies which estimated costs associated with co-morbidities related to CRC or side effects of cancer treatment, studies referring to the cost of CRC screening, studies comparing costs associated with different cancer treatment protocols, and conference proceedings. While the aim of our study was to extract the direct medical costs associated with CRC treatment, we also accepted studies that included cost of illness prior to diagnosis in their definition for CRC treatment, as many countries lack standard screening protocols, resulting in increased spending prior to pathological confirmation of diagnosis. Three authors (RG, VC, VV) independently reviewed the titles and abstracts of articles identified from literature search and resolved discrepancies by consensus. Abstracts that met our inclusion criteria were forwarded for full-text review. The same three authors independently assessed articles eligible for a full-text review based on the inclusion criteria. To assess included studies for quality, we used a modified version of the Consolidated Health Economic Evaluation Reporting Standards (CHEERS) checklist [8], which includes elements that assess for both study and reporting quality and has been used in prior cost of illness systematic reviews [9] (Table S4). We defined studies that scored 14 or greater points on the modified CHEERS checklist as studies of ‘good quality’.

### Data abstraction and synthesis

To characterize the included studies, we extracted information on country, data source, length of follow-up, cancer site (i.e., colon, rectum), sex, sample size, and age-cut off used for estimating and reporting costs. Of particular interest in our systematic review, we extracted detailed information on: 1) costing approach, such as source of payment (i.e., public health spending, private health insurance, out-of-pocket costs); 2) payer perspective (i.e., societal, healthcare provider); 3) whether costs were attributable to (i.e., the mean difference in cost of care between individuals with cancer and without, also referred to as net costs) or associated (i.e., all-cause costs incurred after a CRC diagnosis, which may include the cost of CRC treatment and co-existing conditions) with CRC; and 4) cost components (e.g., chemotherapy, radiation, outpatient visits, inpatient care, and prescription medications). We then extracted the reported per-capita direct medical costs for both prevalent (i.e., existing and newly diagnosed patients) and incident cases of yCRC, and where relevant, aCRC. Also, where relevant, we extracted information on cost comparisons between yCRC and aCRC cases, specifically those based on the use of multivariable approaches. For brevity in our reporting of results, we use the term ‘costs’ to refer to direct medical costs. To facilitate comparisons, all extracted costs were inflation-adjusted to 2020 USA dollars (USD) using the Consumer Price Index unless otherwise specified. Costs in the original currency are provided in Table S5.

## Results

### Literature search results

**Fig. 1:**
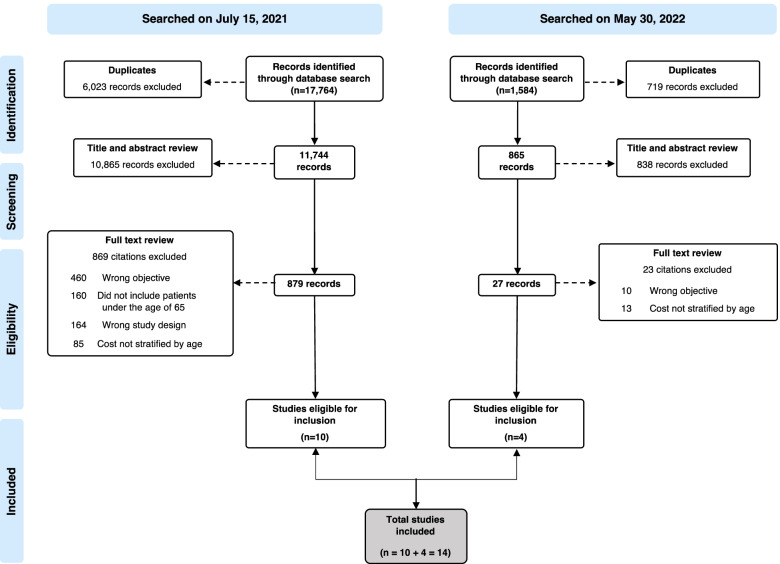
PRISMA Flowchart

Our search strategy resulted in 17,764 articles on July 15, 2021 and 1,584 articles in the May 30, 2022 update (Fig. 1). Article assessment led to exclusions for the following reasons: did not report direct medical costs; did not include patients under the age of 65; and did not report costs stratified by age. Overall, 14 studies met all eligibility criteria and were included in the systematic review – 10 from the original search and 4 from the update.

### Study characteristics

Characteristics of studies included in the systematic review are shown in Table 1. We grouped studies according to those of prevalent (*n* = 5) [10–14] or incident disease (*n* = 9) [15–23]. Studies varied in countries where they were conducted, which included the USA [11, 17, 19], England [15, 18], France [16], China [12, 20], Korea [10], Vietnam [13], Italy [14], Australia [21], Canada [22] and Japan [23]. Five studies utilized claims data from a health insurance database [10, 16, 17, 19, 23], three used data abstracted from hospital medical records [12, 13, 20], four used administrative health data [14, 15, 18, 22], one study used data from a nationally representative medical expenditure survey [11], lastly another study linked self-reported survey data with an administrative health database [21]. It must also be noted that studies which relied on either survey data [11, 21] or hospital medical records [12, 13, 20], may omit data from non-responders and those receiving care from other hospital(s), respectively, which may impact the generalizability of their cost estimates. Most studies estimated the cost of CRC, except for three studies which reported cost estimates by cancer site. Specifically, studies by Gigli and Taplin et al., focused on rectal [14] and colon [17] cancer, respectively, while Goldsbury et al., stratified cost estimates by age and cancer site (i.e., colon and rectum) [21]. CRC was identified using International Disease Classification codes in 10 studies [10, 13, 14, 16–19, 21–23], two studies used hospital medical records [15, 20], and two studies relied on self-reported diagnosis [11, 12]. With respect to cancer stage at diagnosis, 10 studies included CRC patients with stage I to IV disease [12–18, 20, 22, 23], two studies did not report cancer stage [10, 11], one study included CRC patients with a stage IV de novo (no prior CRC diagnosis) diagnosis or a recurrent (previously diagnosed with stage I-III CRC) cancer diagnosis [19], and one study reported cancer severity by the extent of cancer metastasis (i.e., localized, regional, distant metastases) [21]. Overall, nine studies [12, 14, 15, 18–23] scored well against the modified CHEERS checklist with scores ≥ 14 and thus being classified as studies of ‘good quality’ (Table S4). Items not consistently reported by studies that scored below 14 points on the modified CHEERS checklist [10, 11, 13, 16, 17] included, specification of their study perspective (i.e., authors failed to specify their costing perspective), detailed methods for how they estimated resource utilization and costs components, the price date and conversion rate used, and provision of study parameters (i.e., values, references and ranges for input values used to estimate cost).

**Table 1:** Characteristics of included studies on direct medical costs of yCRC

Study	Country	Data Source	Sample size	Female (%)	Age (mean)	Follow-up (years)	Cancer Information			Modified CHEERS score^a^
							**Site**	**Definition**	**Stage**	
**Studies estimating costs of prevalent disease**										
Byun, 2014 [10]	Korea	Claims Health Insurance Review and Assessment Service	83,594	40.7	Not stated	1	Colon Rectal	ICD-10	Not stated	13
Zheng, 2016 [11]	USA	Medical Expenditure Panel Survey	540	55.8	Not stated	1	Colon Rectal	self-report	Not stated	12
Huang, 2017 [12]	China	Hospital medical records; patient survey	2,356	42.9	57.4	Not specified	Colon Rectal	self-report	I-IV	14
Tran, 2020 [13]	Vietnam	Hospital medical records	531	41.4	55.9	1	Colon Rectal	ICD-18,19,20	I-IV	11
Gigli, 2021 [14]	Italy	National Health Service Databases: Cancer registry, Outpatient services, Drug prescriptions, Hospital discharges	9,358	43.0	Not stated	1	Rectal	ICD9-CM	I-IV	14
**Studies estimating costs of incident disease**										
Taplin, 1995 [17]	USA	Health Electronic Records from Cancer Research Network’s Virtual Data Warehouse	1,315	Not stated	Not stated	1	Colon	ICD-O	CIS – distant	12
Clerc, 2008 [16]	France	Public healthcare plan enrollees and health insurance database	204	49.4	Not stated	1	Colon Rectal	ICD-O	I-IV	10
Hall, 2015 [15]	England	Patient Level Information and Costing Systems	146	49.0	65.0	1.5	Colon Rectal	medical records	Dukes staging (A-D)	14
Laudicella, 2016 [18]	England	National Cancer Data Repository; Hospital Episode Statistics; National Schedules of Reference Costs	275, 985	41.5	55.6	9	Colon Rectal	ICD-10	I-IV	14
Ritzwoller, 2018 [19]	USA	Health Electronic Records from Cancer Research Network’s Virtual Data Warehouse	De novo^b^: 1,072 Recurrent^c^: 542	De-Novo^b^: 49.0 Recurrent^c^: 46.0	De-Novo^b^: 66.1 Recurrent^c^: 68.2	1	Colon Rectal	ICD-9,10	De-Novo^b^: IV Recurrent^c^:I-IV	15
Shi, 2019 [20]	China	Hospital medical records	14,536	42.7	58.2	Not specified	Colon Rectal	medical records	I-IV	15
Goldsbury, 2021 [21]	Australia	Sax Institute’s NSW 45 and Up Study questionnaire data linked with administrative datasets, including Admitted Patient Data Collection, Emergency Department Data Collection, NSW Cancer Registry, Pharmaceutical Benefits Scheme and Medicare Benefits Schedule	1,747	Colon: 52.0 Rectal: 35.0	**Median age:** Colon: 72 Rectal: 68	1	Colon Rectal	ICD-10	Localized, regional, distant metastases	16
Paszat, 2021 [22]	Canada	ICES database	9,977	40.5	Not stated	1	Colon Rectal	ICD-10	I-IV	14
Utsumi, 2021 [23]	Japan	National health insurance claims data	Endoscopic^d^: 267 Surgical^e^: 175 Palliative^f^: 161	Endoscopic^d^: 42.7 Surgical^e^: 39.4 Palliative^f^: 34.8	**Median age:** Endoscopic^d^: 67 Surgical^e^: 67 Palliative^f^: 66	3	Colon Rectal	ICD-10	I-IV	14

^a^Modified Consolidated health economic evaluation reporting standards (CHEERS) checklist

^b^De novo CRC patient defined as those without a prior cancer diagnosis

^c^Reccurent CRC patient defined as those previously treated for early-stage (I-III) CRC

^d^Patients who received endoscopic treatment only

^e^Patients who received radical surgery for resection of the primary tumor with or without adjuvant chemotherapy

^f^Patients who were considered to have non-curable CRC

### Costing methodologies

Table 2 highlights aspects of the costing methodology employed by each included study. Given our particular interest in those with yCRC, we synthesized age cut-offs applied, which varied from < 50 years in five studies [10, 13, 14, 22, 23], < 55 years in three studies [12, 20, 21], and < 65 years in six studies [11, 15–19]. While all studies reported costs of CRC treatment (i.e., chemotherapy and radiation therapy) and in-patient visits (i.e., hospitalization), only seven of the included studies considered costs related to ambulatory care, including both outpatient medical visits and prescription medications [10–12, 14, 16, 17, 23]. Additionally, studies varied in terms of the selected costing perspective, with five studies adopting a societal perspective (i.e., considering both patient out-of-pocket costs and health provider spending) [10–13, 20] and nine studies adopting a health provider perspective, of which six estimated public payer spending [14, 15, 18, 21–23] and three utilized data from private payers, including insurance companies [16, 17, 19].

**Table 2:** Methodologies and direct medical costs associated of yCRC versus aCRC

Study	Cost definition	Cost Components					Source of payment			Payer perspective	Cost per-capita CRC (2020 USD)	Cost per-capita aCRC (2020 USD)	Cost comparison (yCRC versus aCRC)	
		Chemotherapy	Radiation Therapy	Outpatient (visits)	Prescription medications	Inpatient (care)	Patient out-of-pocket	Private insurance	Public payer				**Statistical method used**	**Findings**
**Studies estimating the annualized costs of prevalent disease**														
Byun, 2014 [10]	Estimated costs associated with CRC. Only considered the cost of prescription medication if it was indicated for CRC treatment	●	●	●	●	●	●		**●**	Societal^a^	**Male**^b^ 10–19 years: 16,078 20–29 years: 12,466 30–39 years: 10,156 40–49 years: 9,778 **Female**^b^ 10–19 years: 16,801 20–29 years: 15,816 30–39 years: 14,044 40–49 years: 13,658	**Male**^b^ 50–59 years: 9,501 60–69 years: 8,430 70–79 years: 7,580 80–89 years: 6,649 90–100 years: 5,962 **Female**^b^ 50–59 years: 12,148 60–69 years: 11,099 70–79 years: 11,530 80–89 years: 12,466 90–100 years: 12,509	N/A	N/A
Zheng, 2016 [11]	Estimated the costs associated with and attributed to (i.e., net cost) a CRC diagnosis	●	●	●	●	●	●	●	●	Societal^a^	** < 65 years** **Total**^c^**:** 13,837 Outpatient: 7,021 Inpatient: 5,053 Medications: 1,595 **Net**^d^**:** 9,745 Outpatient: 5,597 Inpatient: 3,948 Medications: 650	** ≥ 65 years** **Total**^c^**:** 14,997 Outpatient: 4,151 Inpatient: 5,942 Medications: 3,104 **Net**^d^**:** 5,537 Outpatient: 1,668 Inpatient: 2,970 Prescription: 726	N/A	N/A
Huang, 2017 [12]	Estimated costs associated with CRC. Including 2 months prior to diagnosis until the survey date	●	●	●	●	●	●	●		Societal^a^	** < 45 years**: 11,918 **45–54 years**: 11,994	**55–64 years**: 10,748 ** ≥ 65 years**: 10,644	Generalized linear models	Not significant
Tran, 2020 [13]	Estimated costs associated with CRC	○	○			●				Societal	** < 30 years**: 2,263 **30–39 years**: 2,517 **40–49 years**: 2,335	**50–59 years**: 2,191 **60–69 years**: 1,932 ** ≥ 70 years**: 1,412	N/A	N/A
Gigli, 2021[14]	Estimated cost of care related to rectal cancer treatment (i.e., cost calculation only included the cost of procedures and medications related to rectal cancer treatment)	●	●	●	●	●			●	Healthcare provider	**15–49 years:** Initial^e^: 27,692 Continuing^f^: 3,709 Terminal^g^: 31,359	**50–69 years:** Initial^e^: 24,083 Continuing^f^: 3,290 Terminal^g^: 25,450 **70–79 years:** Initial^e^: 23,184 Continuing^f^: 2,444 Terminal^g^: 18,338 ** ≥ 80 years:** Initial^e^: 18,214 Continuing^f^: 1,620 Terminal^g^: 7,311	N/A	N/A
**Studies estimating the costs of incident disease**														
Taplin, 1995 [17]	Estimated costs associated with colon cancer by phases of care	●	●	●	●	●		●		Healthcare provider^a^	** < 65 years** Initial^e^: 28,013 Continuing^f^: 2,594 Terminal^g^: 30,046	**65–79 years** Initial^e^: 27,614 Continuing^f^: 2,454 Terminal^g^: 22,179 ** ≥ 80 years** Initial^e^: 26,802 Continuing^f^: 2,253 Terminal^g^: 19,575	Multivariate linear regression	Not significant
Clerc, 2008 [16]	Estimated costs associated with CRC during the 12 months following diagnosis	●	●	●	●	●		●		Healthcare provider^a^	** < 65 years:** 46,624^ h^ Medical purchases^i^: 12,101 Outpatient: 8,278 Inpatient: 24,988	**65–74 years:** 42,978^ h^ Medical purchases^i^: $11,949 Outpatient: 7,485 Inpatient: 22,489 ** ≥ 75 years**: 38,668^ h^ Medical purchases^i^: 7,492 Outpatient: 7,076 Inpatient: 23,192	Multivariate linear regression	Not significant
Hall, 2015 [15]	Estimated costs associated with CRC, 6, 12 and 15 months following diagnosis	●	●	●		●			●	Healthcare provider^i^	** < 65 years** 6 months: 19,097 12 months: 23,368 15 months: 25,319	** ≥ 65 years** 6 months: 17,858 12 months: 19,929 15 months: 20,978	Multivariate linear regression	Not significant
Laudicella, 2016 [18]	Estimated costs associated with CRC treatment	●	●			●			●	Healthcare provider^i^	** < 65 years** **Stage 1/2** Year 1: 27,360 Year 2: 6,708 Year 3: 5,631 Year 4: 4,435 Year 5: 4,028 Year 6: 2,873 Year 7: 2,972 Year 8: 2,756 Year 9: 2,428 **Stage 3/4:** Year 1: 35,206 Year 2: 11,774 Year 3: 8,163 Year 4: 6,734 Year 5: 4,910 Year 6: 4,169 Year 7: 4,798 Year 8: 3,763 Year 9: 2,701	** ≥ 65 years** **Stage 1/2:** Year 1: 26,048 Year 2: 6,640 Year 3: 5,567 Year 4: 4,771 Year 5: 4,829 Year 6: 4,872 Year 7: 4,503 Year 8: 4,901 Year 9: 4,229 **Stage 3/4:** Year 1: 28,277 Year 2: 9,437 Year 3: 7,459 Year 4: 6,006 Year 5: 5,668 Year 6: 5,420 Year 7: 3,739 Year 8: 4,629 Year 9: 3,769	N/A	N/A
Ritzwoller, 2018 [19]	Estimated costs associated with CRC during the 12 months following diagnosis	●	●			●		●		Healthcare provider^a^	** < 65 years** De Novo^j^: 89,945 Recurrent^k^: 61,935	** ≥ 65 years** De Novo^j^: 67,195 Recurrent^k^: 49,279	N/A	N/A
Shi, 2019 [20]	Estimated costs associated with CRC treatment	●	●			●	●	●		Societal^a^	**2002–2011** < 45: 7,151 45–54: 7,085 **2009–2011** < 45: 10,087 45–54: 9,846	**2002–2011** 55–64: 6,680 ≥ 65: 6,933 **2009–2011** 55–64: 8,193 ≥ 65: 8,354	N/A	N/A
Goldsbury, 2021[21]	Estimated costs associated with colon and rectal cancer during the 12 months following diagnosis	●	●		●	●			●	Healthcare provider	**45–54 years** Colon: 36,064 Rectal: 40,720	**55- 64 years** Colon: 31,869 Rectal: 36,488 **65–74 years** Colon: 29,723 Rectal: 30,754 ** ≥ 75 years** Colon: 23,251 Rectal: 32,695	Multivariable gamma regression	**Colon:** higher costs associated with age < 55 years old at diagnosis **Rectal:** higher costs associated with age < 65 years old at diagnosis
Paszat, 2021 [22]	Estimated costs associated with CRC during the 12 months following diagnosis	●	●	●		●			●	Healthcare provider	**20–49 years** 44,291	**65–74 years** 41,063	N/A	N/A
Utsumi, 2021[23]	Estimated costs associated with CRC within 3 years of diagnosis	●	●	●	●	●			●	Healthcare provider	**30–39 years** Endoscopic^l^: 8,733 Surgical^m^: 22,755 Palliative^n^: 55,713 **40–49 years** Endoscopic^l^: 5,717 Surgical^m^: 26,386 Palliative^n^: 72,016	**50–59 years** Endoscopic^l^: 8,184 Surgical^m^: 26,507 Palliative^n^: 80,516 **60–69 years** Endoscopic^l^: 12,681 Surgical^m^: 33,424 Palliative^n^: 78,840 **70–71 years** Endoscopic^l^: 12,811 Surgical^m^: 29,160 Palliative^n^: 70,936	N/A	N/A

^a^Extrapolated payer prescriptive based on source of costing data

^b^Calculated based on number of participants

^c^Costs associated with a CRC diagnosis, including all-cause costs incurred after diagnosis

^d^Net cost defined as the difference in healthcare spending among CRC cases and cancer-free controls

^e^Initial phase of care, defined as ≤ 6 months after CRC diagnosis

^f^Continuing phase of care, defined at the time in between initial and terminal phase of care

^g^Terminal phase of care, defined as ≤ 6 months before all-cause mortality

^h^Includes cost of transportation to and from medical appointments, accounts for 2.5% of total cumulative medical expenditure

^i^Includes prescription medications, prothesis and ‘expensive’ chemotherapy agents

^j^De novo CRC patient defined as those without a prior cancer diagnosis

^k^Reccurent CRC patient defined as those previously treated for early-stage (I-III) CRC

^l^Patients who received endoscopic treatment only

^m^Patients who received radical surgery for resection of the primary tumor with or without adjuvant chemotherapy

^n^Patients who were considered to have non-curable CRC

Open dot (○) indicates uncertainty with regards to inclusion of said component in the study

### Direct medical costs of prevalent yCRC

Table 2 summarized key results, namely reported costs for yCRC and where relevant, aCRC. Five studies of prevalent disease reported the annualized per-capita cost for existing as well as new cases of CRC [10–14]. Four out of the five aforementioned studies estimated the annualized per-capita cost associated with CRC (i.e., all-cause costs incurred after a CRC diagnosis, which may include the cost of CRC treatment and co-existing conditions) [10–13]. Specifically, the annualized per-capita cost associated with yCRC ranged from $2,263 to $16,801 (inflation-adjusted to 2020 USD) and $1,412 to $14,997 for aCRC (costs in original currency are provided in Table S5). Among studies estimating the prevalence based cost of CRC, Byun et al. used administrative health data in Korea to account for the cost of treatment (i.e., chemotherapy, radiation and outpatient/inpatient visits) and prescription medications related to a CRC diagnosis (i.e., costs for prescription medications unrelated to CRC treatment were excluded), with estimated per-capita costs associated with yCRC ranging from $9,778 to $16,078 and $13,658 to $16,801 among males and females, respectively. [10]. Inspection of costs stratified by sex and age categories (i.e., 10–19, 20–29, 30–39, 40–49 years) suggest higher costs among females and younger age groups. Next, Zheng et al., used the Medical Expenditure Panel Survey in the US and estimated costs associated with yCRC to be $13,837 and the net cost of illness (or attributable costs, defined as costs of CRC cases minus cancer-free controls) to be $9,745 [11]. Here, net costs accounted for 70% of the total cost of illness incurred by individuals with yCRC. This contrasts to those with aCRC where net costs ($5,537) accounted for 37% of the total cost of illness ($14,997) [11]. Authors also stratified the overall reported cost by cost components (i.e., outpatient/inpatient visits and prescription medications), with outpatient visits accounting for 57% ($5,597) of the net cost for yCRC, in comparison to 30% ($1,668) of the net cost for aCRC patients [11]. Additionally, it must be noted that the yCRC age cut-off applied by Zheng et al., is 65 years [11], while remaining studies employed age cut-offs of < 50 [10, 13, 14] or 55 years old [12]. Using hospital medical records and patient-reported survey data, Huang et al., used generalized linear models to compare the cost of yCRC and aCRC [12]. Specifically, they demonstrated that costs did not significantly differ for those diagnosed at less than 45 (*p* = 0.419), 45–54 (*p* = 0.131) and 55–64 (*p* = 0.522) years old compared to those diagnosed at 65 (reference) years or older [12]. Among prevalence-based, cost estimates by Tran et al., in Vietnam, were the lowest – ranging from $2,263 to $2,517 and $1,412 to $2,191 for yCRC and aCRC, respectively. Low estimation of costs associated with CRC may be attributed to the use of medical expenditure data from a single hospital site. Additionally, authors did not specify whether they included costs for radiation and chemotherapy in their cost estimates, as they simply stated that ‘cancer treatment’ were included in their calculations [13].

Among more recent studies, Gigli et al., used administrative health data to estimate costs directly related to rectal cancer treatment (i.e., cost calculation only considered the cost of procedures and medications used for rectal cancer treatment) by phases of care [14]. Specifically, among young-onset rectal cancer patients costs were estimated to be $27,692, $3,709 and $31,359 during the initial (first 12 months of treatment), continuing (the months between the continuing and terminal phase) and terminal (last 12 months before death) phases of care, respectively [14]. Here, authors found the cost of hospitalizations to be the main driver of the total cost estimate for each phase of care. Cost estimates followed a decreasing trend with age, with average-age onset rectal cancer treatment costs ranging from $18,214 to $24,083, $1,620 to $3,290, and $7,311 to $25,450 during the initial, continuing and terminal phases of care, respectively [14].

### Direct medical costs of incident yCRC

Nine studies estimated the per-capita cost of incident CRC cases, with differences between the selected time horizon, point of care (i.e., treatment phase, continuing phase) and cancer site (i.e., colon or rectum). Of note, in contrast to prevalence-based costing studies that reported age cut-offs consistent with current definitions of yCRC (i.e., cut-offs at 45, 50 years), the majority (*n* = 5) of incidence-based costing studies used an age cut-off of less than 65 years old [15–19].

Five out of the nine included studies captured costs incurred 12 months following a CRC diagnosis, with reported per-capita costs ranging from $23,368 to $89,945 and $19,929 to $67,195 among yCRC and aCRC, respectively [15, 16, 18, 19, 22]. Using data from a health insurance database in France, Clerc et al., stratified their cost estimates by the different healthcare components and reported that 54% ($24,988) and 52% ($22,489) of overall costs incurred during the first 12 months following a CRC diagnosis to be attributed to inpatient hospital visits, among yCRC and aCRC respectively [16]. Additionally, the study employed a multivariate linear regression model to evaluate the impact of covariates on the cost associated with CRC and found no significant association with age at diagnosis [16]. Next, using electronic health records from a cancer registry in the US, Ritzwoller et al., estimated the cost associated with de novo metastatic (stage IV) cancer to be $89,945 and $67,195 among yCRC and aCRC, respectively [19]. Paszat et al., conducted a cohort study in Ontario, Canada using administrative health data and estimated the cost CRC among individuals with a hereditary CRC syndrome to be $44,291 and $41,063 among yCRC and aCRC patients, respectively [22]. Lastly, studies by Hall et al. and Laudicella et al., used an extended time horizon and captured costs beyond 12 months after diagnosis [15, 18]. Specifically, with the use of administrative health data in England, Hall et al., estimated the cost associated with yCRC to be $19,097, $23,368 and $25,319 at 6, 12 and 15 months post-diagnosis, respectively [15]. Authors also used multivariate linear regression models, which demonstrated that age at diagnosis did not significantly impact cost associated with CRC care [15]. Similar results are seen by Laudicella et al., who used administrative health data in England and stratified costs by stage at diagnosis. Here authors extended the time horizon to 9 years since diagnosis, with the highest cost being incurred during the first 12 months after diagnosis [18]. Cost estimates stratified by stage of diagnosis, indicate a greater difference in the cost of illness between early and late stage diagnosis among yCRC (stage I/II $27,360; stage III/IV $35,206; difference -$7,846), as this trend was less pronounced among aCRC patients (stage I/II $26,048; stage III/IV 28,277; difference -$2,229) [18].

Shi et al., used hospital medical records in China to examine trends in the cost of CRC treatment over time (time horizon unspecified) [20]. Here, authors found the costs associated with CRC become increasingly more expensive over the years. Particularly among individuals with yCRC, as the per-capita cost between 2009 and 2011 was estimated to be $9,846 compared to $7,085 between 2002 and 2011 among yCRC patients less than 45 years old. However, this trend was less pronounced among aCRC patients greater or equal to 65 years old (2002–2011, $6,933; 2009–2011, $8,354) [20].

Utsumi et al., used national health insurance claims data in Japan and estimated costs associated with CRC stratified by age and treatment type [23]. Specifically, among yCRC patients the mean cost of care ranged from $5,717 to $8,733 for those who received endoscopic treatment, $22,755 to $26,386 for those who received surgery, and $55,713 to $72,016 for those who received palliative care (i.e., consider non-curable CRC) [23]. In contrast to the aforementioned studies, cost estimates followed an increasing trend with age at diagnosis, as the mean cost of care ranged from $8,184 to $12,811, $26,507 to $33,424 and $70,936 to $80,516 among aCRC patients who received endoscopic, surgical and palliative care, respectively. The low cost of care for patients who received endoscopic treatment, which relates to a lower stage at diagnosis relative to the surgical and palliative treatment groups (indicating late stage diagnosis) among both yCRC and aCRC patients, emphasizes the cost-benefits of early stage diagnosis and treatment.

Lastly, two out of the nine included studies stratified cost estimates by age and cancer site [17, 21]. Taplin et al., used electronic health records from a cancer registry in the USA to estimate the cost of colon cancer at different stages of care among young-onset patients. Specifically, costs were estimated to be $28,013 $30,046 and $2,594 during the initial (first 6 months of treatment), terminal (last 6 months before death) and continuing (the months between the continuing and terminal phase) phases of care, respectively [17]. Meanwhile, Goldsbury et al., linked survey data to an administrative health database and estimated the cost associated with CRC, stratified by both age and cancer site [21]. Specifically, they estimated the cost of colon cancer to be $36,064 and $23,251 to $31,869 among young-onset and average-age onset patients, respectively [21]. In comparison to colon cancer, there was a greater cost associated with rectal cancer, estimated to be $40,720 and ranged from $30,754 to 36,488 among young-onset and average-age onset patients, respectively. Additionally, use of multivariable gamma regression models demonstrated costs estimates to be greater among those diagnosed with colon cancer between the age of 45 and 54 years (effect size 1.10, 95% confidence interval [CI] 1.00–1.12), compared to those diagnosed at the age of 65–74 (reference) [21]. Among rectal cancer patients, a greater estimated cost of care was observed among those diagnosed between the ages of 45–54 (effect size 1.15, 95% CI 1.03–1.28) and 55–65 (Effect size 1.09, 95% CI 1.00–1.18), compared to those diagnosed at the age of 65–74 (reference) [21].

## Discussion

In light of the increasing risk of yCRC [3, 24, 25], we aimed to synthesize evidence on direct medical costs associated with this disease as reported in the 14 included studies in our systematic review. Studies were conducted in 10 countries with different healthcare systems and applied various approaches to costing the direct medical expenditure incurred after a CRC diagnosis, including differing time horizons, data sources and consideration of cost components, all of which led to substantial variation in cost estimates. Among included studies, the annualized per-capita cost of prevalent cases of yCRC ranged from $2,263 to $16,801 (inflation-adjusted to 2020 USD), which provides a snapshot of global healthcare spending on yCRC. Whereas, per-capita costs incurred 12 months following a yCRC diagnosis ranged from $23,368 to $89,945. The costs of incident yCRC provide an estimate of healthcare spending on cancer treatment, which is primarily driven by the cost of chemotherapy, radiation and inpatient care. The majority of studies that evaluated the impact of age of diagnosis did not report statistically significant differences in the costs of yCRC and aCRC. Indeed, an economic burden of yCRC that is similar to aCRC represents substantial impact in the context of increasing risk of yCRC [3, 24, 25], and lends to the ongoing discussions regarding the potential benefits of earlier screening, along with the need for increasing education and awareness for yCRC.

To our knowledge, direct medical costs associated with yCRC have not been systematically evaluated. While Yarboff et al., conducted a systematic review of studies that estimated the economic burden of CRC in 2013, the authors largely focused on evaluating costing methodologies of the included studies [26]. Specifically, they found included studies, even when conducted within the same country, varied in their use of data source, patient population, types of medical services included in their cost calculations and study methodology used to estimate the cost of CRC. These differences across included studies led to substantial variation in cost estimates, and reinforces the need for consistency when reporting patient characteristics, methods and cost estimates in future studies, which will facilitate the comparison of cancer spending across jurisdictions [26]. Perhaps a reflection of the time when this prior systematic review was conducted, they did not provide age-stratified cost estimates, which precluded extrapolation to yCRC. Similar to the systematic review by Yarboff et al., we also report substantial heterogeneity in the costing methodologies adopted by included studies, suggesting the need for consistency or standardization of the approach to estimating and reporting direct medical expenditure. Indeed, when we assessed the quality of included studies, while the majority scored well on the modified CHEERS checklist, many studies did not specify their study perspective (this information was extrapolated based on data source used to estimate costs for a majority of the included studies), and a detailed description of the various components included in their cost estimates. However, in order to inform resource allocation, it is essential for a cost of illness studies to specify the perspective (i.e., who is spending the money?) as well as cost components.

Aside from synthesizing reported costs, observed trends across included studies have implications for better understanding of yCRC. For example, as demonstrated by findings from Ritzwoller et al., and Laudicella et al., costs associated with late stage (stage III/IV) or metastatic CRC were particularly pronounced among yCRC patients, with Rizwoller et al., reporting the cost associated with a metastatic cancer diagnosis to be $89,945 among yCRC patients, compared to $67,195 among aCRC [18, 19]. Additionally, findings by Laudicella et al. demonstrate a more pronounced difference in healthcare expenditure between stage I/II (early stage) and stage III/IV (late stage) among yCRC (-$7,846), compared to aCRC patients (-$2,229) [18]. These findings may also be due to the use of more aggressive treatments such as multi-target chemotherapy regimens, tumor resections and radiotherapy among yCRC patients [27]. Aside from cancer stage, cancer site may also contribute to differences in costs and is likely driven by differences in treatment approaches. Although included studies reported costs associated with differing cancer site (e.g., colon, rectum), the majority did not further stratify those costs by age at diagnosis. Nonetheless, a recent study by Goldsbury et al., reported higher costs associated with rectal cancer in comparison to colon cancer [21]. These findings are of particular relevance given the contribution of rectal cancers to the increasing risk of yCRC [24]. Overall, these results demonstrate the need for future studies to stratify costs associated with yCRC by stage and cancer location to further elucidate the impact of age at diagnosis on healthcare spending and potential cost savings associated with yCRC asymptomatic screening.

While the aim of our study was to capture costs for yCRC, which has largely been defined among adults diagnosed with CRC before the age of 50 years [28], due to limited availability of studies that provide cost estimates by patient subgroups (i.e., age at diagnosis) we considered studies that stratified reported costs based on a cut-off of 65 years and defined individuals diagnosed at less than 65 years of age as ‘young-onset’ for purposes of our systematic review. However, given the rising incidence of yCRC and recent studies indicating a marked increase in CRC cases as individuals shift from 49 to 50 years of age [24, 25], which occurs prior to the age of asymptomatic screening for many countries, it becomes prudent to estimate the cost of CRC at more frequent age intervals (i.e., < 45 and < 50) to inform the need for lowering the age of CRC screening. For example, in Canada, the topic of lowering the age of colorectal cancer screening to 45 year old (currently 50 years old) is highly debated [29]. While experts agree that given the rising incidence of yCRC the expansion of screening protocols may improve mortality outcomes, they are uncertain whether this may translate to a cost benefit due to the opportunity cost incurred by the increased demand for screening tests (i.e., colonoscopy and fecal immunochemical test) [29]. Therefore, to evaluate the cost–benefit of lowering the age of CRC screening it is critical to estimate the cost of yCRC diagnosis, defined as those less than 50 years old. Specifically, this information will have implications for future economic analysis which may model the economic impact of lowering the age of CRC screening to 45 years old.

The strengths and limitations of our systematic review warrant discussion. To ensure a thorough literature search, we developed our search strategy in collaboration with an information scientist who executed all database searches. An original and updated search further ensures comprehensive and timely capture of relevant studies to date. While we standardized all costs to 2020 USD to facilitate interpretation, we caution comparison of costs estimates across different jurisdictions due to differences in the delivery and cost of healthcare services, which also prohibited us from pooling the data (i.e., meta-analysis). As the focus of our study was on direct medical costs of yCRC, we did not synthesize indirect costs such as productivity loss, which is relevant given that as young adults, individuals with yCRC comprise a greater majority of the work-force. As mentioned earlier, for the purposes of our systematic review we considered individuals diagnosed with CRC younger than 65 years old as ‘young-onset’. As such our synthesis may not accurately represent our target population of younger adults diagnosed with CRC.

In conclusion, synthesis of available evidence suggests that the per-capita costs of yCRC is substantial and does not significantly differ from the per-capita costs of aCRC. Given the global rise in the incidence of yCRC [3] and evidence that individuals with yCRC are more frequently diagnosed with late stage disease [5], an economic burden of yCRC that is similar to aCRC represents substantial healthcare spending. However, given the identified limitations in the current literature, it is necessary for future studies to estimate the direct medical expenditure associated with yCRC at ages less than 50 years old and to further stratify cost estimates by stage at diagnosis and cancer site to further elucidate impact of these characteristics on healthcare spending. These cost considerations will be particularly relevant, given expansion of screening strategies to include those less than 50 is a current policy question in many countries [29, 30].

## Supplementary Information

**Additional file 1:** **Supplementary Table-S1.** Database(s): Ovid MEDLINE(R) and Epub Ahead of Print, In-Process & Other Non-Indexed Citations, Daily and Versions(R) 1946 to July 13, 2021. **Supplementary Table-S2.** Database(s): Embase 1974 to July 14, 2021. **Supplementary Table-S3.** Database(s): Web of Science, Science Citation Index and Social Science Citation Index only. **Supplementary Table-S4.** Quality assessment of included studies. **Supplementary Table-S5.** Direct medical costs associated of yCRC versus aCRC, original currency and inflation adjusted to 2020 USD.

## Data Availability

All data generated or analysed during this study are included in this published article and its supplementary information files.

## Abbreviations

CRC: Colorectal Cancer
aCRC: Average age onset Colorectal Cancer
yCRC: Young onset Colorectal Cancer
CHEERS: Consolidated Health Economic Evaluation Reporting Standards
USD: USA Dollars
