# Supplementary material for: Direct medical costs of young-onset colorectal cancer: a worldwide systematic review
Source: BMC Health Serv Res. 2022 Aug 30;22:1100. doi: 10.1186/s12913-022-08481-6 (PMC9426038; doi:10.1186/s12913-022-08481-6)
Supplement: Supplementary file 1 — Additional file 1: Supplementary Table-S1. Database(s): Ovid MEDLINE(R) and Epub Ahead of Print, In-Process & Other Non-Indexed Citations, Daily and Versions(R) 1946 to July 13, 2021. Supplementary Table-S2. Database(s): Embase 1974 to July 14, 2021. Supplementary Table-S3. Database(s): Web of Science, Science Citation Index and Social Science Citation Index only. Supplementary Table-S4. Quality assessment of included studies. Supplementary Table-S5. Direct medical costs associated of yCRC versus aCRC, original currency and inflation adjusted to 2020 USD. [file 12913_2022_8481_MOESM1_ESM.docx]

| **Supplementary Table-S1.** Database(s): Ovid MEDLINE(R) and Epub Ahead of Print, In-Process & Other Non-Indexed Citations, Daily and Versions(R) 1946 to July 13, 2021. | | | |
| --- | --- | --- | --- |
| **Line** | **Searches** | **Results** | |
| 1. | exp Colorectal Neoplasms/ | 212146 | |
| 2. | ((colorectal or colon or rect*) adj3 (cancer* or neoplasm*)).mp. [mp=title, abstract, original title, name of substance word, subject heading word, floating sub-heading word, keyword heading word, organism supplementary concept word, protocol supplementary concept word, rare disease supplementary concept word, unique identifier, synonyms] | 228798 | |
| 3. | 1 or 2 | 270666 | |
| 4. | economics/ | 27346 | |
| 5. | exp "Costs and Cost Analysis"/ | 247209 | |
| 6. | Economics, Nursing/ | 4005 | |
| 7. | Economics, Medical/ | 9139 | |
| 8. | Economics, Pharmaceutical/ | 2998 | |
| 9. | exp Economics, Hospital/ | 25209 | |
| 10. | exp "Fees and Charges"/ | 30801 | |
| 11. | exp Budgets/ | 13853 | |
| 12. | budget*.ti,ab,kf. | 31724 | |
| 13. | (economic* or cost or costs or costly or costing or price or prices or pricing or pharmacoeconomic* or pharmaco-economic* or expenditure or expenditures or expense or expenses or financial or finance or finances or financed).ti,kf. | 245700 | |
| 14. | (economic* or cost or costs or costly or costing or price or prices or pricing or pharmacoeconomic* or pharmaco-economic* or expenditure or expenditures or expense or expenses or financial or finance or finances or financed).ab. /freq=2 | 319262 | |
| 15. | (cost* adj2 (effective* or utilit* or benefit* or minimi* or analy* or outcome or outcomes)).ab,kf. | 177610 | |
| 16. | (value adj2 (money or monetary)).ti,ab,kf. | 2615 | |
| 17. | exp models, economic/ | 15704 | |
| 18. | economic model*.ab,kf. | 3600 | |
| 19. | markov chains/ | 15098 | |
| 20.  21. | markov.ti,ab,kf.  monte carlo method/ | 24612  29878 | |
| 22.  23.  24. | monte carlo.ti,ab,kf.  exp Decision Theory/  (decision* adj2 (tree* or analy* or model*)).ti,ab,kf. | 52817  12571  27743 | |
| 25.  26. | or/4-24  3 and 25 | 782754  6256 | |
| 27. | limit 26 to (english or french) | 5923 | |
| 28. 27 and (202107* or 202108* or 202109* or 20211* or 2022*).dt,ez,da. 625  **Supplementary Table-S2.** Database(s): Embase 1974 to July 14, 2021. | | |  |
| **Line** | **Searches** | **Results** |  |
| 1. | exp *colon cancer/ | 169445 |  |
| 2. | exp *rectum cancer/ | 150897 |  |
| 3. | ((colorectal or colon or rect*) adj3 (cancer* or neoplasm*)).tw,kw. | 278719 |  |
| 4. | 1 or 2 or 3 | 315162 |  |
| 5. | economics/ | 241554 |  |
| 6. | cost/ | 59514 |  |
| 7. | exp health economics/ | 891159 |  |
| 8. | budget/ | 30666 |  |
| 9. | budget*.ti,ab,kw. | 42043 |  |
| 10. | (economic* or cost or costs or costly or costing or price or prices or pricing or pharmacoeconomic* or pharmaco-economic* or expenditure or expenditures or expense or expenses or financial or finance or finances or financed).ti,kw. | 303063 |  |
| 11. | (economic* or cost or costs or costly or costing or price or prices or pricing or pharmacoeconomic* or pharmaco-economic* or expenditure or expenditures or expense or expenses or financial or finance or finances or financed).ab. /freq=2 | 447404 |  |
| 12. | (cost* adj2 (effective* or utilit* or benefit* or minimi* or analy* or outcome or outcomes)).ab,kw. | 249862 |  |
| 13. | (value adj2 (money or monetary)).ti,ab,kw. | 3566 |  |
| 14. | economic model*.ab,kw. | 5358 |  |
| 15. | or/5-14 | 1404982 |  |
| 16. | 4 and 15 | 13211 |  |
| 17. | limit 16 to (english or french) | 12676 |  |
| 18. | limit 17 to "humans only (removes records about animals)" | 12611 |  |
| 19. | limit 18 to (conference abstracts or medline) | 5259 |  |
| 20. | 18 not 19 | 7352 |  |
| 21. | Limit 20 to dc=20210715-20220530 | 609 |  |

| **Supplementary Table-S3.** Database(s): Web of Science, Science Citation Index and Social Science Citation Index only | | |
| --- | --- | --- |
| **Line** | **Searches** | **Results** |
| 1. | (TI=(((colorectal or colon or rect*) NEAR/3 (cancer* or neoplasm*)))) OR AB=(((colorectal or colon or rect*) NEAR/3 (cancer* or neoplasm*))) | 202,442 |
| 2. | (TI=(economic* or cost or costs or costly or costing or price or prices or pricing or pharmacoeconomic* or pharmaco-economic* or expenditure or expenditures or expense or expenses or financial or finance or finances or financed)) OR AB=(economic* or cost or costs or costly or costing or price or prices or pricing or pharmacoeconomic* or pharmaco-economic* or expenditure or expenditures or expense or expenses or financial or finance or finances or financed) | 2,386,204 |
| 3. | (TI=(cost* NEAR/2 (effective* or utilit* or benefit* or minimi* or analy* or outcome or outcomes)) OR AB=(cost* NEAR/2 (effective* or utilit* or benefit* or minimi* or analy* or outcome or outcomes))) | 324,822 |
| 4. | (TI=((value NEAR/2 (money or monetary)))) OR AB=((value NEAR/2 (money or monetary))) | 5,897 |
| 5. | #2 OR #3 OR #4 | 2,388,985 |
| 6. | #1 AND #5 | 6,327 |
| 7. | #1 AND #5 and Articles (Document Types) and English or French (Languages) | 4,489 |

Supplementary Table-S4. Quality assessment of included studies.

|  | Byun *et al.*, 2014 | Zheng *et al.*, 2016 | Huang *et al.*, 2017 | Tran *et al.*, 2020 | Taplin *et al.*, 1995 |
| --- | --- | --- | --- | --- | --- |
| Title | 1 | 1 | 1 | 1 | 1 |
| Abstract | 1 | 1 | 1 | 0 | 1 |
| Background and objectives | 1 | 1 | 1 | 1 | 1 |
| Target population and subgroups | 1 | 1 | 1 | 1 | 1 |
| Setting and location | 1 | 1 | 1 | 1 | 1 |
| Study perspective | 0 | 0 | 0 | 1 | 0 |
| Population |  |  |  |  |  |
| Time horizon | 1 | 1 | 1 | 1 | 1 |
| Cost components | 1 | 1 | 1 | 0 | 1 |
| Estimating resources and costs | 1 | 0 | 0 | 0 | 0 |
| Currency, price date, and conversion | 1 | 1 | 1 | 0 | 1 |
| Analytical methods | 0 | 0 | 0 | 0 | 0 |
| Study parameters | 0 | 0 | 0 | 0 | 0 |
| Cost | 0 | 1 | 1 | 1 | 1 |
| Characterising uncertainty | 0 | 1 | 1 | 0 | 0 |
| Characterising heterogeneity | 1 | 1 | 1 | 1 | 1 |
| Study findings, limitations, generalisability, and current knowledge | 1 | 1 | 1 | 1 | 1 |
| Source of funding | 1 | 0 | 1 | 1 | 1 |
| Conflicts of interest | 1 | 0 | 1 | 1 | 0 |
| Total | 13 | 12 | 14 | 11 | 12 |

|  | Clerc *et al.*, 2008 | Hall *et al.*, 2015 | Laudicella *et al.*, 2016 | Ritzwoller *et al.*, 2018 | Shi *et al.*, 2019 |
| --- | --- | --- | --- | --- | --- |
| Title | 1 | 1 | 1 | 1 | 1 |
| Abstract | 0 | 1 | 1 | 1 | 1 |
| Background and objectives | 1 | 1 | 1 | 1 | 1 |
| Target population and subgroups | 1 | 1 | 1 | 1 | 1 |
| Setting and location | 1 | 1 | 1 | 1 | 1 |
| Study perspective | 0 | 0 | 0 | 1 | 0 |
| Population |  |  |  |  |  |
| Time horizon | 1 | 1 | 1 | 1 | 1 |
| Cost components | 1 | 1 | 1 | 1 | 1 |
| Estimating resources and costs | 0 | 1 | 1 | 1 | 0 |
| Currency, price date, and conversion | 0 | 0 | 0 | 0 | 1 |
| Analytical methods | 0 | 1 | 1 | 1 | 1 |
| Study parameters | 0 | 0 | 0 | 0 | 0 |
| Cost | 1 | 1 | 1 | 1 | 1 |
| Characterising uncertainty | 1 | 1 | 0 | 1 | 1 |
| Characterising heterogeneity | 0 | 1 | 1 | 1 | 1 |
| Study findings, limitations, generalisability, and current knowledge | 1 | 1 | 1 | 1 | 1 |
| Source of funding | 1 | 1 | 1 | 1 | 1 |
| Conflicts of interest | 0 | 0 | 1 | 1 | 1 |
| Total | 10 | 14 | 14 | 16 | 15 |

|  | Gigli *et al.*, 2021 | Goldsbury *et al*., 2021 | Paszat *et al.*, 2021 | Utsumi *et al.*, 2021 |  |
| --- | --- | --- | --- | --- | --- |
| Title | 1 | 1 | 1 | 1 |  |
| Abstract | 1 | 1 | 1 | 1 |  |
| Background and objectives | 1 | 1 | 1 | 1 |  |
| Target population and subgroups | 1 | 1 | 1 | 1 |  |
| Setting and location | 1 | 1 | 1 | 1 |  |
| Study perspective | 1 | 1 | 1 | 1 |  |
| Population |  |  |  |  |  |
| Time horizon | 1 | 1 | 1 | 1 |  |
| Cost components | 1 | 1 | 1 | 1 |  |
| Estimating resources and costs | 1 | 1 | 1 | 1 |  |
| Currency, price date, and conversion | 0 | 1 | 0 | 0 |  |
| Analytical methods | 1 | 1 | 0 | 0 |  |
| Study parameters | 1 | 1 | 0 | 1 |  |
| Cost | 0 | 1 | 1 | 1 |  |
| Characterising uncertainty | 0 | 1 | 1 | 1 |  |
| Characterising heterogeneity | 0 | 1 | 0 | 0 |  |
| Study findings, limitations, generalisability, and current knowledge | 1 | 1 | 1 | 1 |  |
| Source of funding | 1 | 0 | 1 | 0 |  |
| Conflicts of interest | 1 | 0 | 1 | 1 |  |
| Total | 14 | 16 | 14 | 14 |  |

**Supplementary Table-S5.** Direct medical costs associated of yCRC versus aCRC, original currency and inflation adjusted to 2020 USD

| **Study** | **Exchange Rate/ Original Currency** | **Cost per-capita yCRC in original currency** | **Cost per-capita aCRC in original currency** | **Cost per-capita yCRC (2020 USD)** | **Cost per-capita aCRC (2020 USD)** |
| --- | --- | --- | --- | --- | --- |
| Byun, 2014 | 1 Won (2010) = 0.0009 USD | **Male^a^**  10-19 years: 1,5050,000  20-29 years: 11,668,919  30-39 years: 9,506,746  40-49 years: 9,152,582  **Female^a^**  10-19 years: 15,727,273  20-29 years: 14,804,878  30-39 years: 13,146,313  40-49 years: 12,784,933 | **Male^a^**  50-59 years: 8,893,977  60-69 years: 7,891,434  70-79 years: 7,095,096  80-89 years: 6,223,934  90-100 years: 5,581,152  **Female^a^**  50-59 years: 11,371,502  60-69 years: 10,389,802  70-79 years: 10,792,976  80-89 years: 11,669,093  90-100 years: 11,708,955 | **Male^a^**  10-19 years: 16,078  20-29 years: 12,466  30-39 years: 10,156  40-49 years: 9,778  **Female^a^**  10-19 years: 16,801  20-29 years: 15,816  30-39 years: 14,044  40-49 years: 13,658 | **Male^a^**  50-59 years: 9,501  60-69 years: 8,430  70-79 years: 7,580  80-89 years: 6,649  90-100 years: 5,962  **Female^a^**  50-59 years: 12,148  60-69 years: 11,099  70-79 years: 11,530  80-89 years: 12,466  90-100 years: 12,509 |
| Zheng, 2016 | 2012, USD | **<65 years**  **Total^b^:** 12,278  Outpatient: 6,230  Inpatient: 4,484  Medications: 1,415  **Net^c^:** 8,647  Outpatient: 4,966  Inpatient: 3,503  Medications: 577 | **≥65 years**  **Total^b^:** 13,307  Outpatient: 3,683  Inpatient: 5,272  Medications: 2,754  **Net^c^:** 4,913  Outpatient: 1,480  Inpatient: 2,635  Prescription: 644 | **<65 years**  **Total^b^:** 13,837  Outpatient: 7,021  Inpatient: 5,053  Medications: 1,595  **Net^c^:** 9,745  Outpatient: 5,597  Inpatient: 3,948  Medications: 650 | **≥65 years**  **Total^b^:** 14,997  Outpatient: 4,151  Inpatient: 5,942  Medications: 3,104  **Net^c^:** 5,537  Outpatient: 1,668  Inpatient: 2,970  Prescription: 726 |
| Huang, 2017 | 1 Chinese Yan (2014) = 0.163 USD | **<45 years**: 65,887  **45–54 years**: 66,303 | **55-64 years**: 59,416  **≥65 years**: 58,840 | **<45 years**: 11,918  **45–54 years**: 11,994 | **55-64 years**: 10,748  **≥65 years**: 10,644 |
| Tran, 2020 | 1 Vietnam Dong (2018) = 0.00004 USD | **<30 years**: 50,228,000  **30-39 years**: 55,852,000  **40-49 years**: 51,822,000 | **50-59 years**: 48,615,000  **60-69 years**: 42,881,000  **≥70 years**: 31,338,000 | **<30 years**: 2,263  **30-39 years**: 2,517  **40-49 years**: 2,335 | **50-59 years**: 2,191  **60-69 years**: 1,932  **≥70 years**: 1,412 |
| Gigli, 2021 | 1 Euro (2011) = 1.39 USD | **15-49 years:**  Initial: 17,279  Continuing: 2,314  Terminal: 19,567 | **50-69 years:**  Initial: 15,027  Continuing: 2,053  Terminal: 15,880  **70-79 years:**  Initial: 14,466  Continuing: 1,525  Terminal: 11,442  **≥80 years:**  Initial: 11,365  Continuing: 1,011  Terminal: 4,562 | **15-49 years:**  Initial: 27,692  Continuing: 3,709  Terminal: 31,359 | **50-69 years:**  Initial: 24,083  Continuing: 3,290  Terminal: 25,450  **70-79 years:**  Initial: 23,184  Continuing: 2,444  Terminal: 18,338  **≥80 years:**  Initial: 18,214  Continuing: 1,620  Terminal: 7,311 |
| Taplin, 1995 | 1992, USD | **<65 years**  Initial^d^: 15,183  Continuing^e^: 1,406  Terminal^f^: 16,285 | **65-79 years**  Initial^d^: 14,967  Continuing^e^: 1,330  Terminal^f^: 12,021  **≥80 years**  Initial^d^: 14,527  Continuing^e^: 1,221  Terminal^f^: 10,610 | **<65 years**  Initial^d^: 28,013  Continuing^e^: 2,594  Terminal^f^: 30,046 | **65-79 years**  Initial^d^: 27,614  Continuing^e^: 2,454  Terminal^f^: 22,179  **≥80 years**  Initial^d^: 26,802  Continuing^e^: 2,253  Terminal^f^: 19,575 |
| Clerc, 2008 | 1 Euro (2004) = 1.24 USD | **<65 years:** 27445^g^  Medical purchases: 7,123  Outpatient: 4,873  Inpatient: 14,709 | **65-74 years:** 25,299^g^  Medical purchases: 7,034  Outpatient: 4,406  Inpatient: 13,238  **≥75 years**: 22,762^g^  Medical purchases: 4,410  Outpatient: 4,165  Inpatient: 13,652 | **<65 years:** 46,624^g^  Medical purchases: 12,101  Outpatient: 8,278  Inpatient: 24,988 | **65-74 years:** 42,978^g^  Medical purchases: 11,949  Outpatient: 7,485  Inpatient: 22,489  **≥75 years**: 38,668^g^  Medical purchases: 7,492  Outpatient: 7,076  Inpatient: 23,192 |
| Hall, 2015 | 1 Pound (2011) = 1.60 USD | **<65 years**  6 months: 10,343  12 months: 12,656  15 months: 13,713 | **≥65 years**  6 months: 9,672  12 months: 10,794  15 months: 11,362 | **<65 years**  6 months: 19,097  12 months: 23,368  15 months: 25,319 | **≥65 years**  6 months: 17,858  12 months: 19,929  15 months: 20,978 |
| Laudicella, 2016 | 1 Pound (2010) = 1.55 USD | **<65 years**  **Stage 1/2**  Year 1: 14,911  Year 2: 3,656  Year 3: 3,069  Year 4: 2,417  Year 5: 2,195  Year 6: 1,566  Year 7: 1,620  Year 8: 1,502  Year 9: 1,323  **Stage 3/4:**  Year 1: 19,187  Year 2: 6,417  Year 3: 4,449  Year 4: 3,670  Year 5: 2,676  Year 6: 2,272  Year 7: 2,615  Year 8: 2,051  Year 9: 1,472 | **≥65 years**  **Stage 1/2:**  Year 1: 14,196  Year 2: 3,619  Year 3: 3,034  Year 4: 2,600  Year 5: 2,632  Year 6: 2,655  Year 7: 2,454  Year 8: 2,671  Year 9: 2,305  **Stage 3/4:**  Year 1: 15,411  Year 2: 5,143  Year 3: 4,065  Year 4: 3,273  Year 5: 3,089  Year 6: 2,954  Year 7: 2,038  Year 8: 2,523  Year 9: 2,054 | **<65 years**  **Stage 1/2**  Year 1: 27,360  Year 2: 6,708  Year 3: 5,631  Year 4: 4,435  Year 5: 4,028  Year 6: 2,873  Year 7: 2,972  Year 8: 2,756  Year 9: 2,428  **Stage 3/4:**  Year 1: 35,206  Year 2: 11,774  Year 3: 8,163  Year 4: 6,734  Year 5: 4,910  Year 6: 4,169  Year 7: 4,798  Year 8: 3,763  Year 9: 2,701 | **≥65 years**  **Stage 1/2:**  Year 1: 26,048  Year 2: 6,640  Year 3: 5,567  Year 4: 4,771  Year 5: 4,829  Year 6: 4,872  Year 7: 4,503  Year 8: 4,901  Year 9: 4,229  **Stage 3/4:**  Year 1: 28,277  Year 2: 9,437  Year 3: 7,459  Year 4: 6,006  Year 5: 5,668  Year 6: 5,420  Year 7: 3,739  Year 8: 4,629  Year 9: 3,769 |
| Ritzwoller, 2018 | 2012, USD | **<65 years**  De Novo^h^: 79,809  Recurrent^i^: 54,956 | **≥65 years**  De Novo^h^: 59,623  Recurrent^i^: 43,726 | **<65 years**  De Novo^h^: 89,945  Recurrent^i^: 61,935 | **≥65 years**  De Novo^h^: 67,195  Recurrent^i^: 49,279 |
| Shi, 2019 | 1 Chinese Yuan (2011) = 0.159 USD | **2002-2011**  <45: 39,099  45-54: 38,736  **2009-2011**  <45: 55,153  45-54: 53,835 | **2002-2011**  55-64: 36,524  ≥65: 37,908  **2009-2011**  55-64: 44,797  ≥65: 45,678 | **2002-2011**  <45: 7,151  45-54: 7,085  **2009-2011**  <45: 10,087  45-54: 9,846 | **2002-2011**  55-64: 6,680  ≥65: 6,933  **2009-2011**  55-64: 8,193  ≥65: 8,354 |
| Goldsbury, 2021 | 1 Australian dollar (2020) = 0.69 USD. | **45-54 years**  Colon: 52,266  Rectal: 59,014 | **55- 64 years**  Colon: 46,187  Rectal: 52,881  **65-74 years**  Colon: 43,077  Rectal: 44,571  **≥75 years**  Colon: 33,697  Rectal: 47,384 | **45-54 years**  Colon: 36,064  Rectal: 40,720 | **55- 64 years**  Colon: 31,869  Rectal: 36,488  **65-74 years**  Colon: 29,723  Rectal: 30,754  **≥75 years**  Colon: 23,251  Rectal: 32,695 |
| Paszat, 2021 | 1 Canadian dollar (2017) = 0.77 USD | **20-49 years**  54,471 | **65–74 years**  50,501 | **20-49 years**  44,291 | **65-74 years**  41,063 |
| Utsumi, 2021 | 1 Japanese Yen (2019)^I^ = 0.0092 USD | **30-39 years**  Endoscopic: 938,000  Surgical: 2,444,000  Palliative: 5,984,000  **40-49 years**  Endoscopic: 614,000  Surgical: 2,834,000  Palliative: 7,735,000 | **50-59 years**  Endoscopic: 879,000  Surgical: 2,847,000  Palliative: 8,648,000  **60-69 years**  Endoscopic: 1,362,000  Surgical: 3,590,000  Palliative: 8,468,000  **70-71 years**  Endoscopic: 1,376,000  Surgical: 3,132,000  Palliative: 7,619,000 | **30-39 years**  Endoscopic: 8,733  Surgical: 22,755  Palliative: 55,713  **40-49 years**  Endoscopic: 5,717  Surgical: 26,386  Palliative: 72,016 | **50-59 years**  Endoscopic: 8,184  Surgical: 26,507  Palliative: 80,516  **60-69 years**  Endoscopic: 12,681  Surgical: 33,424  Palliative: 78,840  **70-71 years**  Endoscopic: 12,811  Surgical: 29,160  Palliative: 70,936 |

^a^Calculated based on number of participants

^b^Costs associated with a CRC diagnosis, including all-cause costs incurred after diagnosis

^c^Net cost defined as the difference in healthcare spending among CRC cases and cancer-free controls

^d^Initial phase of care, defined as ≤ 6months after CRC diagnosis

^e^Continuing phase of care, defined at the time in between initial and terminal phase of care

^f^Terminal phase of care, defined as ≤ 6months before all-cause mortality

^g^Includes cost of transportation to and from medical appointments, accounts for 2.5% of total cumulative medical expenditure

^h^De novo CRC patient defined as those without a prior cancer diagnosis

^i^Year of reported costs not specified, assumed based on study time period
